# Counseling young women with early breast cancer on fertility preservation

**DOI:** 10.1007/s10815-019-01615-6

**Published:** 2019-11-23

**Authors:** M. E. (Elena) ter Welle-Butalid, I. J. H. (Ingeborg) Vriens, J. G. (Josien) Derhaag, E. M. (Edward) Leter, C. E. (Christine) de Die-Smulders, M. (Marjolein) Smidt, R. J. T. (Ron) van Golde, V. C. G. (Vivianne) Tjan-Heijnen

**Affiliations:** 1 Department of Obstetrics and Gynaecology, Maastricht University Medical Center, P.O. Box 5800, 6202 AZ Maastricht, The Netherlands; 2 GROW - School for Oncology and Developmental Biology, Maastricht University Medical Center, P.O. Box 5800, 6202 AZ Maastricht, The Netherlands; 3 Department of Internal Medicine, division of Medical Oncology, Maastricht University Medical Center, P.O. Box 5800, 6202 AZ Maastricht, The Netherlands; 4 Department of Clinical Genetics, Maastricht University Medical Center, P.O. Box 5800, 6202 AZ Maastricht, the Netherlands; 5 Department of Surgery, Maastricht University Medical Center, P.O. Box 5800, 6202 AZ Maastricht, the Netherlands

**Keywords:** Breast cancer, Chemotherapy, Young women, Fertility preservation, Cryopreservation

## Abstract

**Purpose:**

Women with early-stage breast cancer may still have a future child wish, while chemotherapy may impair fertility. To pursue on fertility preservation shortly after breast cancer diagnosis is complex. This review holds a critical reflection on all topics that need to be counseled to give them the opportunity to make a well-informed decision before starting any oncological treatment.

**Methods:**

A comprehensive literature review was performed on papers published in English language on breast cancer in young women, risk of chemotherapy-induced infertility, fertility preservation techniques, impact of possible mutation carriership, and future pregnancy outcome.

**Results:**

Below 40 years of age, the risk of permanent chemotherapy-induced ovarian function failure is approximately 20%, where taxanes do not significantly add to this risk. Overall, 23% of reported women who performed fertility preservation by cryopreserving oocytes or embryos returned for embryo transfer. Of these, 40% gave live birth. Both fertility preservation in women diagnosed with breast cancer and pregnancy after treatment seem safe with respect to breast cancer survival. Women who have a genetic predisposition for breast cancer like *BRCA* gene mutation should also be informed about the possibility of pre-implantation genetic diagnosis.

**Conclusions:**

Women with an early stage of breast cancer and a possible future child wish should be referred to an expertise center in breast cancer, fertility preservation, and genetics in this complex decision-making process, shortly after diagnosis.

## Introduction

Breast cancer is the most commonly diagnosed malignancy in women [1]. Annually, 180,000 women worldwide are diagnosed with early-stage breast cancer and are younger than 40 years of age [1]. As young age is a poor prognostic factor, (neo)adjuvant chemotherapy is frequently advised in this group of women [2, 3]. Chemotherapy may however induce premature ovarian insufficiency and thus impair fertility [4]. Conversely, increasing survival rates after breast cancer have heightened the importance of quality of life issues. One of these is the ability to have children. Considering the trend towards postponing childbearing until the late reproductive years, the number of childless women when diagnosed with breast cancer will further increase [5].

Currently, young breast cancer patients are given the opportunity to consider fertility preservation. Among the diverse possibilities for fertility preservation, freezing embryos or oocytes during an in vitro fertilization (IVF) procedure for later use is most frequently used [6]. Cryopreserving ovarian tissue is also a possible method, but still under debate whether this technique preserves fertility better as compared with IVF [7]. Besides this, the possible risk of reintroduction of malignant cells is under investigation.

**Fig. 1:**
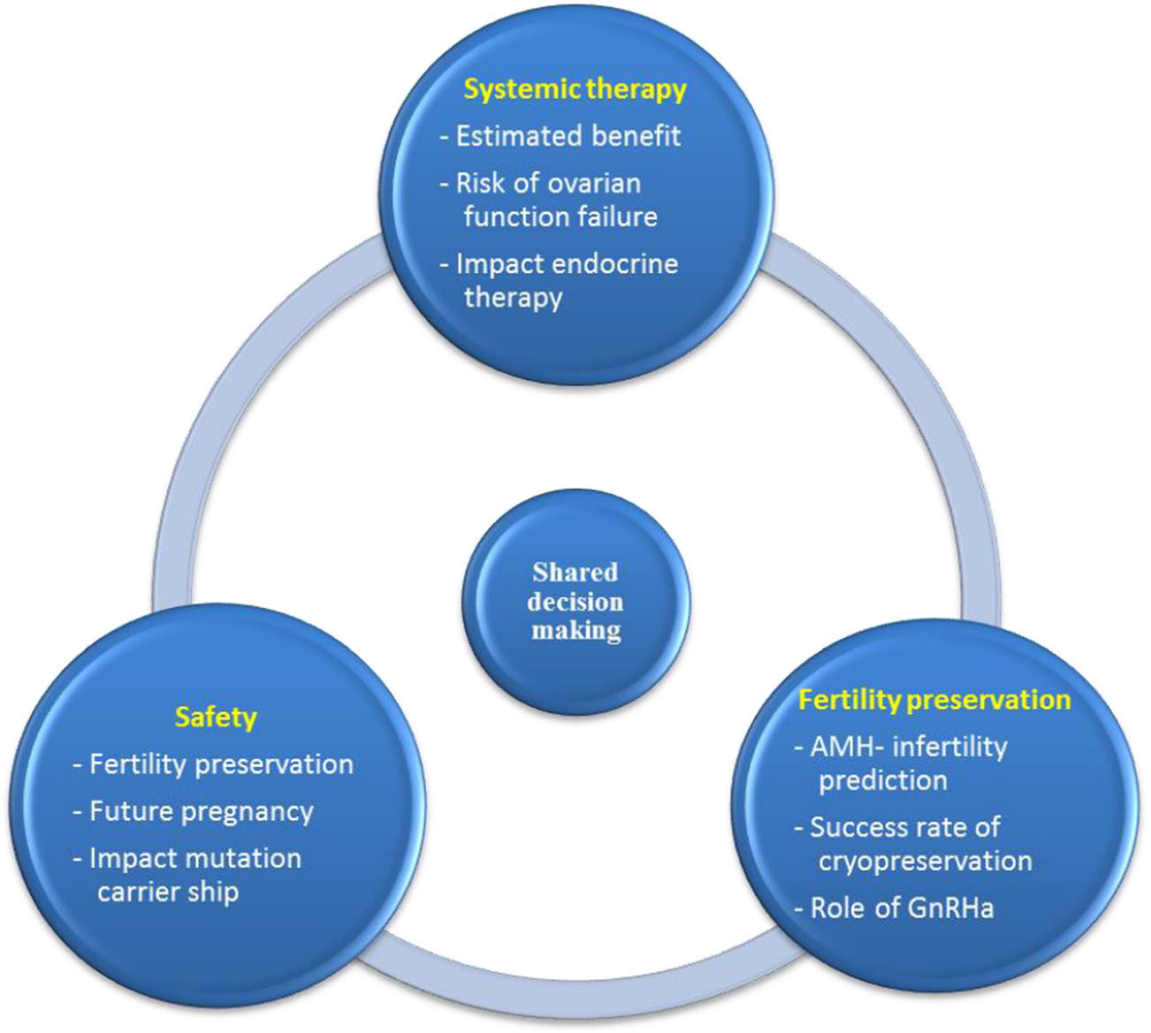
Topics that need to be discussed when counseling young women with early-stage breast cancer and (possible) indication for (neo)adjuvant chemotherapy with respect to fertility

Women with early-stage breast cancer have to decide shortly after breast cancer diagnosis if they want to pursue a fertility preservation procedure [7]. Therefore, it is important to give these women complete counseling to optimize decision-making. This review holds a critical reflection on all topics that need to be counseled. We will elaborate on: the prognosis and estimated benefit of chemotherapy, the risk of chemotherapy-induced ovarian function failure, the impact of adjuvant endocrine therapy, the role of antimullerian hormone assessment in predicting risk of infertility, the success rate of currently used fertility preservation procedures, the value of gonadotropin-releasing hormone analogue (GnRHa) use for preventing ovarian function failure, the safety of the fertility preservation procedure, the safety of pregnancy after breast cancer, and the impact of possible presence of a mutation in one of the genes with a predisposition for hereditary breast cancer on fertility preservation choices (Fig. 1).

## Methods

A comprehensive literature review was performed on papers published in English language on breast cancer in young women regarding risk of chemotherapy-induced ovarian function failure and role of adjuvant endocrine therapy, safety, and effectiveness of fertility preservation techniques, and impact on future pregnancy and breast cancer outcome. These papers were found by multiple searches through the PubMed database and by following cited references. Additionally, updates from the main conferences in the field of oncology, assisted reproduction, and fertility preservation were used.

## Prognosis and estimated benefit of chemotherapy

It is reported that independent of age and tumor characteristics, third-generation chemotherapy regimens reduce breast cancer mortality by one-third [8]. A low absolute risk of recurrence implies a low absolute benefit from chemotherapy and vice versa. The estimated absolute 10-year survival benefit of chemotherapy for the general breast cancer population ranges from 1 to 15%.

Breast cancer in young women has less favorable biological features, including higher histological grade, higher Ki67, and lymphovascular invasion and therefore a higher risk of recurrence. Online nomograms are available for estimating survival rates based on patient and tumor characteristics, but these nomograms do not perform well in the very young [9, 10].

Chemotherapy can cause significant side effects, which may especially be troubling in these young women. They are in the middle of their working, social, and family life [11, 12]. Foremost, chemotherapy may induce premature ovarian insufficiency, leading to postmenopausal symptoms and impair fertility [4].

In conclusion, many young women with early breast cancer diagnosis may substantially benefit from chemotherapy in terms of survival gain, but this has to be balanced against the risk of undesired long-term side effects. Counseling on the possible benefits and harms of chemotherapy, including impact on fertility, is important to optimize decision-making on the indication of chemotherapy.

## Chemotherapy-induced ovarian function failure

Regarding the risk of chemotherapy-induced menopause and risk of infertility, both the type of chemotherapy and the patients’ age are important factors to consider.

Although the mechanisms of chemotherapy-induced ovarian function failure are not entirely understood, Adriamycin and cyclophosphamide are known to cause double-stranded DNA breaks, and thereby apoptosis of primordial follicles and depletion of the follicular pool [13]. Adriamycin further causes vascular and stromal damage in the ovary by an acute reduction in ovarian blood flow and disintegration of the blood vessels, resulting in oxidative stress [13, 14]. Taxanes also cause depletion of the follicular pool but do not cause direct vascular damage [15, 16].

Previously, it was reported that in patients aged 40 years or less, four cycles of Adriamycin-cyclophosphamide (AC) chemotherapy was associated with a low risk (< 20%), cyclophosphamide, methotrexate, 5-fluorouracil (CMF) with a low to an intermediate risk (< 60%), and AC followed by taxane with an intermediate risk (40–60%) of premature menopause [17, 18]. However, this risk classification may need some reconsiderations (see below).

Noteworthy, the moment of developing amenorrhea differs per type of chemotherapy. During CMF chemotherapy, amenorrhea occurs in over half of the patients, with no recovery after the end of chemotherapy and a continuous further rise in amenorrhea to approximately 80% of women after 3 years [19]. Conversely, in nearly all patients menstrual bleedings vanish while receiving AC chemotherapy, yet the ovarian function slowly recovered during the first 9 months after the end of chemotherapy in most of patients [19]. Moreover, in three-quarter of patients with chemotherapy-induced ovarian function failure, premenopausal hormone levels are the first evidence of ovarian function recovery, whereas only in one-quarter resumption of menses is the first sign [20]. Knowledge on these patterns of ovarian function failure and recovery is relevant when assessing the risk of ovarian function failure of different chemotherapy regimens.

Recently, more studies reported the impact of taxanes (Table 1) [20–28]. In most studies, amenorrhea was used as a surrogate for chemotherapy-induced ovarian function failure. However, many patients also received adjuvant tamoxifen. Tamoxifen does not increase the risk of permanent ovarian function failure, but during tamoxifen, the menstrual cycle may be absent [18] [29–31]. Hence, the reported amenorrhea rates overestimate the risk of definitive ovarian function failure. Interestingly, from these recent studies, the earlier-mentioned increased risk of chemotherapy-induced amenorrhea by taxanes could not be confirmed. On average 20% of patients below 40 years of age (range 9–29%) still show ovarian function failure 1 year after the end of taxane-based, third-generation chemotherapy (Table 1).

**Table 1:** Incidence of chemotherapy-induced amenorrhea 12 months after the end of chemotherapy for early-stage breast cancer, classified by the use of taxanes and by age

First author	Year	No. patients by chemotherapy regimen: without vs. with taxane	No. patients by age < 40 vs. ≥ 40 years	Rate of incidence of chemotherapy-induced amenorrhea	
				Impact of taxane independent of age (no vs. yes)	Impact of age independent of taxane (< 40 vs. ≥ 40 years)
Addition of taxanes					
Abusief [21]	2010	228 (4AC) 204 (4AC–4T)	138 296	55 56	*13* 75
Sukumvanich [22]	2010	111 (AC) 160 (AC–T)	254 212	19 29	*13* 54
Yoo [23]	2013	192 (4AC) 120 (4AC–4T)	104 208	64 54	*18* 77
Replacement of 5FU by taxanes in combination or sequential schedules					
Narmadha [24]	2012	36 (6FAC/6FEC) 14 (6TAC/6TEC)	21 29	48 64	*26* 69
Berliere^a^ [25]	2008	84 (6FEC) 70 (3FEC–3T)	39 115	76 64	*29* 79
Han^a^ [26]	2009	129 (6FAC) 34 (4AC–4T)		72 74	–
Zhou [27]	2012	85 (6FEC) 80(3FEC–3T/4EC–4T/6TEC)	64 101	49 38	*11* 64
Okanami [28]	2011	17 (6CAF) 49 (4AC–4T/6CAF–4T/6FEC–4T)	66 0	12 25	–
Vriens^a^ [20]	2017	19 (6FEC) 96 (4AC–4T/6TAC)	23 90	67 79	*9* 86

*A* Adriamycin, *C* cyclophosphamide, *E* epirubicin, *F* 5-fluorouracil, *T* taxane (Docetaxel or Paclitaxel)

^a^In these studies, FSH and E2 measurements were performed

p<0.05

In conclusion, with the currently used chemotherapy regimens, the risk of permanent chemotherapy-induced ovarian function failure is on average 20% in patients below 40 years of age. This risk is not increased when taxanes are added to the AC chemotherapy regimen.

## The use of adjuvant endocrine therapy

In patients with low-risk hormone receptor-positive breast cancer, 5 years of tamoxifen is considered a standard therapy. For high(er) risk hormone receptor-positive breast cancer patients, prolonged endocrine treatment for 10 years in combination with ovarian function suppression may be considered, in addition to chemotherapy [32, 33].

Tamoxifen does not increase the risk of permanent ovarian function failure, but due to its action, the menstrual cycle may be absent while using tamoxifen [18, 29–31]. Ovarian function should be monitored when tamoxifen is used after chemotherapy, because unnoticed ovarian function recovery while taking tamoxifen may lead to teratogenicity in unplanned pregnancies [34]. The use of adequate non-hormonal, barrier contraceptive measures should be advised.

In conclusion, hormonal therapies (tamoxifen, aromatase inhibitors, GnRHa) do not have irreversible effects on ovarian function but should be timely interrupted when trying to become pregnant, considering the risk of teratogenicity.

## Use of antimullerian hormone in fertility preservation

During chemotherapy in patients with breast cancer, antimullerian hormone (AMH) levels drop. Low pretreatment AMH levels predict low recovery rates of AMH levels after chemotherapy [35–38]. Therefore, it could be hypothesized that a low AMH value pre-chemotherapy could predict infertility after chemotherapy. As fertility preservation is generally recommended to discuss in women with a future child wish and chemotherapy indication, the key question is whether in these younger women the AMH pre-chemotherapy value has an added value for the prediction of infertility after chemotherapy.

Women with a definitive chemotherapy-induced menopause have significant lower pre-chemotherapy AMH values than in those with recovery of ovarian function within 6 months to 2 years [39–42]. However, it should be mentioned that “definitive chemotherapy-induced menopause” was differently defined between studies. In these studies, the mean age of women was above 40 years of age [39–42]. When evaluating women ≤ 40 years of age, no difference in pre-chemotherapy AMH values between those who did or did not have amenorrhea post-chemotherapy was found [36, 38]. Only one study investigated the relationship between AMH and the occurrence of spontaneous pregnancies, and found that neither baseline nor post-chemotherapy AMH values were associated with the chance of spontaneous pregnancies [43].

On the other hand, AMH is a well-established ovarian reserve test as it is a proven predictive marker for ovarian response during in vitro fertilization (IVF) stimulations [44]. However, AMH seems to be only a weak independent predictor for live birth outcome and individualized dosage of gonadotropins after AMH assessment does not seem to improve live birth rate in IVF stimulations [45–47]. Women with an individualized starting dose after AMH assessment have 55.9% chance of live birth, compared with 58.3% in those receiving a standard dose of 150 IE (*P* = 0.13) [46]. But due to restrictive time to collect oocytes in a fertility preservation procedure, cycle cancellation due to poor response should be avoided. Therefore, standard dosage of gonadotropins in this specific setting might not be the best strategy. To minimalize the risk of poor response, a minimum of 150 IE gonadotropins should be used. The potential risk of ovarian hyperstimulation syndrome is low due to the use of a GnRH antagonist, the possible use of an agonist trigger for ovum pick up, and the absence of an embryo transfer.

We conclude that there is no added value of measuring pre- or post-chemotherapy AMH values in predicting infertility. AMH assessment to optimize dosage of gonadotropins does not seem to increase live birth rates. However, in the fertility preservation setting, cycle cancellation due to poor response should be avoided. Gonadotropin dosage should be sufficient and individualized on AMH or other ovarian response markers.

## Success rate of currently used fertility preservation procedures

Cryopreservation of embryos or oocytes is a well-established technique of fertility preservation [7, 48]. Data on pregnancy and live birth rates after transfer of cryopreserved and thawed oocytes or embryos are limited for this specific patient population. Presumably, at least the same results as obtained in regular IVF can be reached, as these couples are not known with subfertility issues [49].

In Table 2, the results of studies with embryo cryopreservation concerning fertility preservation for different oncology indications are summarized [48, 50–60]. In these studies, the weighted mean number of oocytes retrieved after stimulation was 11.1 (range 8.2–12.4), and the mean number of embryos cryopreserved was 5.8 (range 4.1–6.7). In the studies for which the number of transfers of thawed embryos was reported, 11% of all thawed embryos resulted in a live birth [54–56]. Overall, 23% of 614 women (range 13–63%) underwent one or more embryo transfers. Of these, 40% had a live birth (range 9–75%). Twin pregnancies were reported in 39% (range 18–100%) of the patients [48, 51, 53, 54, 56, 57, 60].

**Table 2:** Overview of studies reporting on embryo cryopreservation, return and live birth rates after fertility preservation with an oncological indication

Author	Freezing period	Number of patients	Oocytes retrieved (mean)	Mean embryos cryopreserved	Patients returned, no. (%)	Patients with positive HCG	Patients with live birth, no. (%)
Michaan et al. [50]	2002–2007	19	8.8		4 (21)	3 or 4	3 (75)
Robertson et al. [51]	2001–2007	38	12	6	10^a^ (26)	6	5 (50)
Babb et al. [52]	1979–2007	8			5^a^ (63)	3	2 (40)
Sabatini et al. [53]	1997–2007	28	11.7		12^a^ (43)	5	3 (25)
Barcroft el al. [54]	1996–2011	39	10.6	6.7	5 (13)	3	2 (40)
Courbiere et al. [55]	1999–2011	52	8.2	4.2	11^b^ (21)	5	3 (27)
Dolmans et al. [56]	1997–2014	52	9.7	4.1	9 (17)	6	4 (44)
Oktay et al.^h^ [57]		131	11.8	5.9	33^a^ (25)	20	17^c^ (52)
Cardozo et al. [60]	1997–2014	57	12.4	6.6	21^a^ (39)	11	9 (43)
Hammarberg et al. [58]	1995–2014	170^d^	11.6	6.4	22^d,e^ (13)	4 (18.2%)	2^f^ (9)
Chien et al.^h^ [48]	2010–2017	20		8.7	6^a^ (30)		1 (17)
Moravek et al. [59]	2005–2015	204^g^		6	19 (9)	11	11 (58)
Weighted mean			11.1	5.8	157 (23)		62 (39)

^a^Gestational carrier was used in 20–50% of the patients

^b^One patient had a complete lysis of the embryo after thawing and had no embryo transfer

^c^One patient had two live births

^d^These includes patients who had embryo cryopreservation, oocyte cryopreservation, and ovarian cortex tissue cryopreservation

^e^Thawing was unsuccessful in six thawing procedures; there were no embryos formed after thawing in 5 thawing procedures

^f^Two still were pregnant at the time of study, one of these was pregnant after transfer of two embryos with one stored before cancer treatment and one fresh embryo created after treatment

^g^This group includes patients who had embryo cryopreservation and oocyte cryopreservation

^h^Studies with only breast cancer patients

The first live birth after cryopreserved oocytes for an oncologic indication was reported in 2007 [61]. The number of patients returning for embryo transfer after prior oocyte preservation varies from 0 to 5% [48, 49, 52, 60, 62–64]. Those who returned had a live birth rate between 33 and 50%.

Data were generally published 1–4 years after freezing of oocytes or embryos. Return rates are also influenced by the general advice to wait at least 2 years after diagnosis before trying to become pregnant [65, 66]. Moravek et al. described that 86% of the women had contacted the hospital within the last year of publication date [59]. This implies that not all women had the possibility to return yet and that the effective return rate likely will be higher with a longer observation time. But, it could also reflect a lower than anticipated need of embryo transfer due to a recovered or maintained ovarian function after the end of chemotherapy or, for example, changed view on family planning.

The twin birth rate as mentioned above seems relatively high. This is a result of multiple embryo transfer in most studies. Twin pregnancies have higher obstetric and neonatal risks [67]. Single embryo transfers should be performed.

When women are single at the time of fertility preservation, oocyte cryopreservation is usually used. Moreover, oocyte cryopreservation may even be preferred in all situations, since both partners have ownership over the cryopreserved embryos introducing difficulties if the relationship ends [68, 69].

Unfortunately, older patients who have a higher risk of permanent chemotherapy-induced ovarian function failure are those with the poorer results with cryopreservation techniques. The ovarian response to stimulation is often lower with fewer oocytes available and oocyte quality is diminished often due to more chromosomal abnormalities. Even though there is no consensus on a definite age limit to propose cryopreservation techniques, both physicians and patients should be informed that the probability of conceiving using oocytes vitrified after 35 are rapidly declining [70, 71].

Ovarian cryopreservation is another technique for fertility preservation, still considered experimental but advancing quickly, and may evolve to become a standard approach in the future [7, 72–74]. A recent update on all published papers worldwide reported the results from transplantation of ovarian tissue in 318 women from 21 different countries [75]. Cancer diagnosis was available in 264, and in 24%, breast cancer was diagnosed. Of 237 women, the ovarian function after transplantation was reported, shown to be restored in 95% of the cases. Of all 318 women undergoing transplantation, 170 cases primarily aimed to restore fertility. Transplantation resulted in live births in 69 women, in half of them spontaneous pregnancy was obtained (orthotopic transplants). Of these women, 84% was postmenopausal before transplantation. One-third achieved live birth after IVF. As discussed in the updated ASCO guideline on fertility preservation, ovarian tissue cryopreservation does not require ovarian stimulation and consequently does not cause any delays in oncological treatment [7]. However, ovarian tissue transplantation may bear the risk of reintroducing tumor cells, especially in breast cancer, even though so far the results up until now are re-assuring. The optimization of isolation techniques of ovarian follicles from cryopreserved ovarian tissue and optimizing successful in vitro ovarian follicle maturation may minimize this risk of reintroducing malignant cells in the future. However, in women with hormone receptor positive disease, transplantation of ovarian tissue may—to a greater extent than cryopreserved oocytes—interfere with the aim of ovarian function suppression to improve breast cancer outcome. In addition, the yield of ovarian tissue transplantation with respect to number of live births is not higher than that of cryopreserved oocytes or embryos. Furthermore, for pre-implantation genetics (PGD) in mutation carriers, IVF is still required. Hence, more studies in breast cancer are required to determine its exact place in future.

In conclusion, cryopreservation of embryos or oocytes is a well-established technique of fertility preservation. It should be considered to prefer oocyte cryopreservation over embryo cryopreservation, due to the ethical difficulties that can rise once a relationship is broken or once one of both partners deceases. About half of the collected oocytes will develop into an embryo, irrespective of the moment of fertilization (before or after cryopreservation). The reported return rates for embryo transfer are low (23%), probably as a result of a short follow-up period. Further analysis of non-returning patients is needed. Although the data on live birth rate after cryopreservation are limited, the results are encouraging. Of women returned, on average 40% had at least one live birth. The data suggest that for breast cancer patients, the live birth rates are even higher [57]. However, higher age (> 35 years) is predictive of poor outcome to conceive with vitrified oocytes and should be considered in counseling.

Ovarian cryopreservation is an alternative option that may be considered if it is not possible to perform an ovarian stimulation for oocyte or embryo cryopreservation, which in daily practice is seldom the case.

## Gonadotropin-releasing hormone analogues for gonadal protection

Prevention of ovarian depletion could be a better approach to prevent infertility than preserving fertility by freezing oocytes, embryos, or even ovarian tissue. The use of gonadotropin-releasing hormone analogues (GnRHa) has been hypothesized as an agent used for gonadal protection [7]. Unfortunately, up until now, the actual mechanism of GnRHa in possible gonadal protection is not fully understood [76].

In a recent update of the ASCO guideline on fertility preservation by Oktay and colleagues, seven randomized controlled trials evaluating GnRHa use during chemotherapy were discussed [7]. In the most recent meta-analyses by Lambertini et al., five of these trials with a total of 873 patients were included with a median follow-up of 1.6–7.3 years [77]. The primary endpoint of these studies was ovarian function failure defined by absence of menstrual activity 1–2 years after the start of chemotherapy, in some studies, supported by estradiol and FSH assessments. One-third of patients were aged above 40 years. The meta-analysis showed that in patients with available data on ovarian function, 14% developed ovarian failure in the GnRHa group versus 31% in the control group. Of note, two-third of patients received adjuvant tamoxifen which may have suppressed the menstrual cycle. Thus, the actual ovarian failure rates may be lower.

The POEMS trial is the only trial reporting on number of pregnancies as a preplanned secondary end point [78]. They found a pregnancy rate of 23.1% in the GnRHa group versus 12.2% in the control group (*P* = 0.04) [79]. Lambertini et al. summarized data from five trials and reported at least one post-treatment pregnancy in 10.3% of 359 women in the GnRHa group versus in 5.5% of 367 in the control group (*P* = 0.018) [77]. The added appendix data showed the respective live birth rates of 5.8% versus 2.7% (*P* = 0.043). These findings should be interpreted with great care considering the missing data and non-adjustment for pregnancy desire.

The ASCO guideline states: “There is conflicting evidence to recommend gonadotrophin-releasing hormone agonists (GnRHa) and other means of ovarian suppression for fertility preservation. The Panel recognizes that, when proven fertility preservation methods are not feasible, and in the setting of young women with breast cancer, GnRHa may be offered to patients in the hope of reducing the likelihood of chemotherapy-induced ovarian insufficiency. GnRHa should not be used in place of proven fertility preservation methods.” [7]

In line with this statement, we conclude that patients interested in reducing the risk of post-treatment amenorrhea and menopausal symptoms may choose this approach. However, it is important to inform patients on the controversy and uncertainty regarding the efficacy of GnRHa as a “fertility preservation” approach.

## Safety of fertility preservation

Breast cancer could theoretically be stimulated by the temporally hyperestrogenic state during an in vitro fertilization (IVF) procedure in the context of fertility preservation, and an early state of hormone-sensitive breast cancer might disseminate. After a regular IVF procedure, there is no increased risk to develop breast cancer compared with women who did not undergo IVF [80], although women who had IVF seem to have a transient increase in the risk of having breast cancer (hazard ratio (HR) = 1.96) diagnosed in the first year after treatment [81]. To reduce the possible deleterious hyperestrogenic effect, letrozole or tamoxifen are recommended during the ovarian hyperstimulation in patients with a recent breast cancer diagnosis [82–84]. But, whether this improves safety remains unclear [85, 86].

So far, there are no signs of a higher recurrence rate of breast cancer in woman who opt for a fertility preservation procedure after breast cancer diagnosis, although the follow-up period of most studies is short [87–89]. In these studies, the tumor was generally removed before the fertility preservation procedure. Currently, chemotherapy is increasingly offered as neoadjuvant treatment to patients with early breast cancer. Studies on the safety of performing the fertility preservation procedure while the primary tumor is still in situ are however sparse [48]. Patients have to be counseled about the timing of chemotherapy, discussing both the pros and cons from an oncological viewpoint and the lack of data on the safety of performing the fertility preservation procedure while the tumor is still present. Our team recommends primary surgery in patients with hormone receptor-positive disease, whereas both options (adjuvant and neoadjuvant chemotherapy) are available for patients with hormone receptor negative disease.

The urgency to continue oncological treatment and perform fertility preservation in a short period of time is inconvenient when deciding on which stimulation protocol is needed. Recent literature shows that fertility preservation can start randomly in a menstruation cycle, without a negative effect on the number of oocytes retrieved [7].

In conclusion, fertility preservation in women diagnosed with breast cancer seems safe with the use of tamoxifen or letrozole. Fertility preservation before neoadjuvant chemotherapy is probably safe in patients with hormone receptor negative tumors, although follow-up data are very sparse. In patients with hormone receptor-positive disease, we recommend to first perform breast surgery. With current procedures, fertility preservation can start randomly in a menstruation cycle, hence not causing a significant delay in oncological treatment.

## Safety of pregnancy after breast cancer

Several studies have shown that pregnancy does not negatively impact breast cancer prognosis [66, 90–92]. In a large European study, it was questioned whether pregnancy was also safe for women with a prior hormone receptor-positive breast cancer [90]. In their study with a matched-control design including 686 patients with hormone receptor-positive disease, they showed no significant difference in disease-free survival for the pregnant versus non-pregnant women. The pregnant group showed even a better overall survival (HR = 0.72; *P* = 0.03), with no interaction according to hormone receptor status.

Three population-based studies have been performed on birth outcome in women treated for breast cancer [93–95]. Their findings indicate that a breast cancer history may correspond with 50% increase in risk of delivering a preterm birth and low birth weight compared with the general population, with greater increases in risk observed among women who received chemotherapy or gave birth within 2 years of diagnosis.

In conclusion, pregnancy after breast cancer does not seem to impact the risk of breast cancer recurrence. Pregnancy may result more often in a preterm birth and lower birth weight (relative increase of 50%) as compared with the general population.

## Fertility preservation and counseling in (*BRCA*) mutation carriers

The *BRCA1* or *BRCA2* mutation detection rate in female breast cancer patients depends on factors such as age at diagnosis and breast cancer subtype. Of women diagnosed before the age of 45 years with a positive family history, 12.0% carried *BRCA1* or *BRCA2* germline mutations [96]. Mutations in other breast cancer predisposition genes are even more rare. Testing for breast cancer related genes is recommended in women below the age of 50 [97].

Hereditary predisposition for breast cancer might influence the decision to choose for a fertility preservation procedure. For *BRCA1* and *BRCA2* mutation carriers, the knowledge that risk-reducing salpingoopherectomy is recommended between 35–40 and 40–45 years of age respectively, may add to the decision to choose for fertility preservation [98]. The possibility to use pre-implantation genetic diagnosis (PGD) for hereditary breast cancer in the future could be another reason. In PGD, embryos obtained by an IVF procedure are tested for the presence of the specific mutation, and only embryos without the mutation will be transferred into the uterus. PGD on previously cryopreserved and thawed embryos or fertilized cryopreserved and thawed oocytes is a fair option. So, when fertility preservation is performed, PGD could be added as a (future) option. In clinical settings, PGD is often mentioned by mutation carriers as the decisive reason to opt for fertility preservation before the start of chemotherapy [99, 100].

*BRCA* mutation carriers can perceive a pressure to fulfill their child wish at an earlier age, for example due to a shortened reproductive life span [101]. Therefore, fertility preservation can also be considered in healthy *BRCA* mutation carriers [102].

Women with an increased risk of hereditary breast cancer may have extra concerns about the safety regarding a fertility preservation procedure. One study compared *BRCA* mutation carries after an IVF stimulation versus no IVF stimulation. Breast cancer incidence was comparable in both groups, therefore, performing an IVF stimulation in these women can be considered safe [103].

Regarding the reproductive decisions a woman has to make, the concerns regarding ovarian reserve in these women should also be taken into account. Specifically, the *BRCA1* gene might contribute to ovarian ageing as it is involved in DNA double-strand break repair [104–106]. However, there are conflicting results on a clinical relevant diminished ovarian reserve. We recently showed the total number of oocytes retrieved is on average one oocyte lower in women with a *BRCA* mutation compared with controls [107]. This difference seems too small to be of clinical relevance.

In conclusion, besides the counseling on fertility preservation options young women with (*BRCA*) gene mutations should be informed about the possibility of PGD. The advice to perform risk-reducing salpingoopherectomy should be counseled as well, since this could also be a reason to perform fertility preservation. There are no concerns regarding safety and oocyte outcome in an IVF procedure in these women.

## Summarizing conclusions and remarks

The conclusions of this critical reflection are summarized in Table 3. Considering the complexity of the entire decision process, we recommend referral to a center with expertise in breast cancer, fertility preservation, and clinical genetics, and with special interest in this specific patient group. Referral should take place shortly after diagnosis of early breast cancer and before final decisions on the oncological treatment plan have been made.

**Table 3:** Summarizing conclusions and remarks

Conclusions	
1	Counseling on the possible benefits and harms of chemotherapy, including impact on fertility, is important for the patient to make a well-informed decision on the initiation of chemotherapy.
2	With the currently used chemotherapy regimens, the risk of permanent chemotherapy-induced ovarian function failure is on average 20% in patients below 40 years of age, with the lowest risk in the very young. Taxanes do not add to the risk of AC chemotherapy.
3	Hormonal therapies (tamoxifen, aromatase inhibitors, GnRHa) do not have irreversible effects on ovarian function but should be timely interrupted when trying to fulfill a child wish, also because of its teratogenicity.
4	The value of pre-chemotherapy AMH values in reliably predicting the chance of a spontaneous pregnancy after chemotherapy is not shown and should not routinely be used.
5	Cryopreservation of embryos or oocytes is a well-established technique of fertility preservation. The reported return rate for embryo transfer is on average 23%. But, of women returned, on average 40% had at least one live birth. Oocyte cryopreservation may be preferred over embryo cryopreservation, due to the practical, psychological, and ethical difficulties that can rise once a relationship is broken or once one of both partners deceases.
6	In line with the ASCO guideline, we conclude that GnRHa should not be considered as a first line fertility preservation method.
7	Fertility preservation in women diagnosed with breast cancer seems safe with the use of tamoxifen or letrozole. In patients with hormone receptor-positive disease, we recommend to first perform breast surgery instead of neoadjuvant chemotherapy. With current procedures, fertility preservation can start randomly in a menstruation cycle, hence not causing a significant delay in oncological treatment.
8	Pregnancy after breast cancer does not seem to impact the risk of breast cancer recurrence. Pregnancy after breast cancer treatment may however result more often in a preterm birth and lower birth weight (relative increase of 50%) as compared with the general population, especially after chemotherapy and pregnancy within 2 years of diagnosis.
9	Besides the counseling on fertility preservation options, young women with (*BRCA*) gene mutations should be informed about the possibility of PGD. The advice to perform risk-reducing salpingoopherectomy should be counseled as well, since this could also be a reason to perform fertility preservation. There are no additional concerns regarding safety and oocyte outcome in an IVF procedure in these women.
10	When women are confronted with infertility after breast cancer and neither gametes nor embryos are preserved or anymore available, counseling on their remaining reproductive options is needed. Couples can opt for alternatives like oocyte donation, adoption, and foster parenthood, or can decide to remain childless.
